# Rivaroxaban as Therapy for Saphenous Venous Graft Failure due to Venous Outflow Mismatch

**DOI:** 10.1155/2022/9729989

**Published:** 2022-03-16

**Authors:** Matthew T. Lee, Ayush Mohan, Jenna E. Lee, Daniel T. Lee

**Affiliations:** ^1^Central Michigan University College of Medicine, USA; ^2^McLaren Bay City Regional Medical Center, USA

## Abstract

**Background:**

Recurrent angina and long-term occlusion following coronary artery bypass graft surgery is often treated with percutaneous coronary intervention, a high-risk intervention for distal embolization. Here, we present the utilization of the novel oral anticoagulant, rivaroxaban, in the treatment of saphenous vein graft thrombosis with complete resolution of the thrombus secondary to graft outflow mismatch. *Case Presentation*. A 69-year-old man with triple coronary artery bypass grafting using a saphenous vein and left internal mammary artery, performed in 2017, presented at our hospital for recurrent angina. Coronary angiography revealed a patent LIMA to LAD and a large clot burden in the venous conduit to the first OM/terminal circumflex—theorized to be due to an outflow mismatch of the large saphenous vein to the native artery resulting in stasis. Instead of percutaneous coronary intervention, he was treated with rivaroxaban 20 mg once a day. The angiography 4 weeks after starting rivaroxaban showed complete resolution of the thrombus.

**Conclusion:**

Rivaroxaban could become a potential treatment option in thrombus reversal due to static venous flow with subsequent long-term patency of the graft. Additionally, its use may be indicated in the generalized prevention of VGF.

## 1. Background

Saphenous vein grafts (SVGs) remain the most used conduit during coronary artery bypass graft (CABG) surgery. However, during the first year after bypass surgery, up to 15% of venous grafts occlude and are prone to long-term occlusion and up to 20% of patients have recurrent angina within the first year [1]. Venous graft failure (VGF) is a multifactorial process that has not been completely understood. The pathogenesis could involve accelerated atherosclerosis, inflammation, thrombosis, or any combination [2]. Graft failure, however, could be from stasis secondary to a mismatch of the venous conduit with the outflow of the native vessel [3]. Herein, we report a case report of a patient who presents NSTEMI. Angiography revealed a large SVG clot burden due to venous outflow mismatch which was subsequently resolved with four weeks of rivaroxaban.

## 2. Case

**Figure 1:**
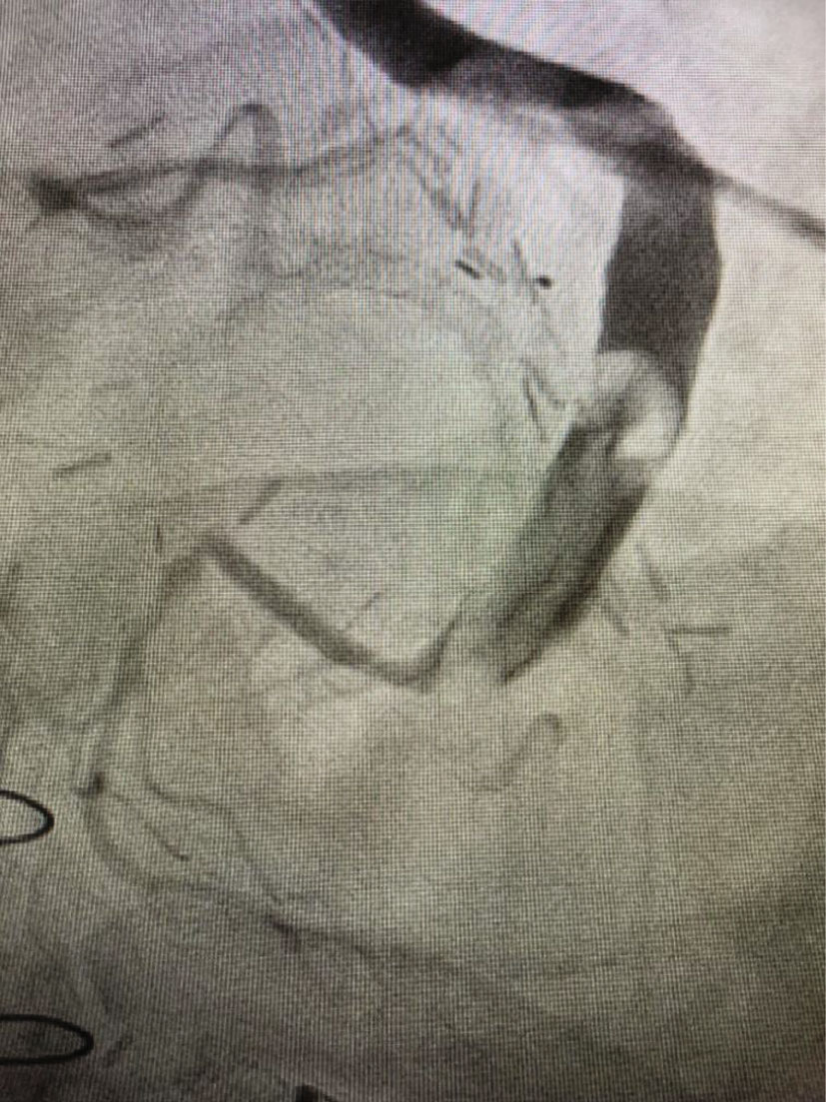
Initial cardiac angiogram of the first OM/terminal circumflex with high clot burden theorized to be due to venous outflow mismatch from SVG.

**Figure 2:**
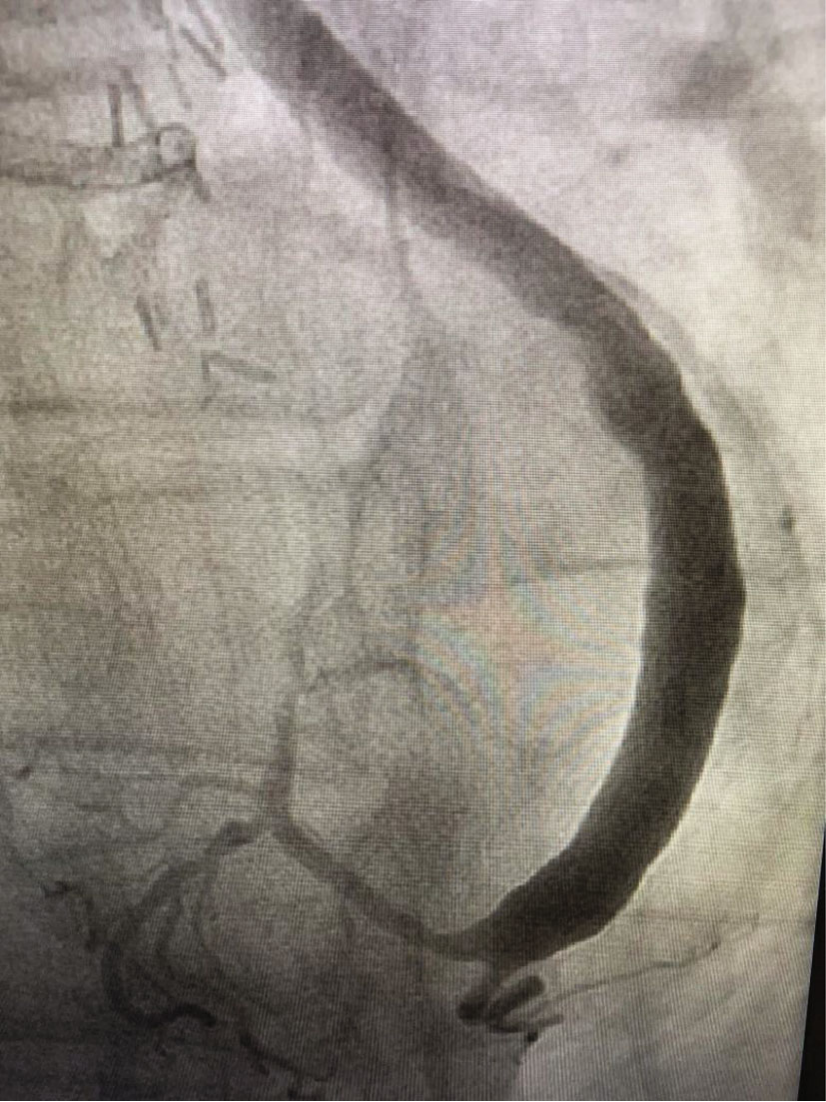
Postrivaroxaban therapy cardiac angiogram of the first OM/terminal circumflex with high clot burden showing complete resolution after four weeks of pharmacotherapy.

A 69-year-old male presents with NSTEMI, with pertinent medical history of triple coronary artery bypass grafting (CABG) from 2017. At that time, revascularization was anatomically complete and included the following grafts: left internal mammary artery (LIMA) to the left anterior descending coronary artery and saphenous vein sequential bypass graft to the first obtuse marginal coronary artery (OM) and the terminal circumflex coronary artery. There were no complications noted during the CABG procedure. Bypass angiography revealed a patent LIMA to LAD and a large clot burden in the venous conduit to the first OM/terminal circumflex (Figure 1), theorized to be due to an outflow mismatch of the large saphenous vein to the native artery resulting in stasis. With the combination of the large clot burden and poor outflow, the operator speculated high likelihood of distal embolization with SVG stenting. Subsequently, conservative management was instituted. Percutaneous intervention and repeat CABG after saphenous vein graft failure were considered but have significantly increased risks of mortality. Conservative management involving anticoagulant therapy given the cause of thrombosis was ultimately decided. Warfarin was not considered due to increased bleeding risk and delayed, unpredictable pharmacodynamic response. Instead, the patient continued aspirin 81 mg/day and added anticoagulation rivaroxaban 20 mg/day. Four weeks later, angiography of the venous conduit showed complete resolution of the thrombus (Figure 2).

## 3. Discussion

CABG remains an integral part of the treatment of ischemic heart disease. It is one of the most frequently performed surgical procedures in the United States, over 400,000 annually. However, the long-term patency remains at 50% degradation of the saphenous venous grafts at 10 years [4]. Standard treatment of degenerated SVG includes stent placement with or without a distal embolic protection device. These high-risk interventions lead to distal embolization with subsequent NSTEMI up to 25% of the time. Atherosclerosis is the main etiology of late VGF, occurring 12 months after the procedure [1]. Our important findings were twofold. The case clearly represented venous graft failure. The likelihood of atherosclerosis was unlikely; subsequently treating this large clot burden with stenting would have significantly increased the risk of distal embolization with subsequent postprocedure MI, with subsequent risk of in-stent restenosis and repeat thrombosis as the venous conduit to small vessel outflow mismatch would persist. This case represented thrombosis due to stasis secondary to venous outflow mismatch. As a result, rivaroxaban therapy was more appropriate to address immediate resolution of the thrombus with subsequent long-term patency of the graft.

Secondly, we have demonstrated the ability of rivaroxaban to engage in complete thrombus reversal due to static venous flow. While the anticoagulant effects of rivaroxaban are used for its improved outcomes in the treatment of many disease processes including but not limited to DVT, PE, and atrial fibrillation [5, 6], there has not been evidence of its use for complete thrombus reversal due to VGF as we have demonstrated here. This thrombus reversal was theorized to be due to rivaroxaban-induced reduction of factor Xa's inhibitor effect on fibrinolysis [7], therefore increasing the effects of endogenous plasmin.

Beyond the effects of factor Xa inhibition, rivaroxaban acts as an antiplatelet agent [8] which further decreases the rate of potential thrombus formation in the setting of SVG. Moreover, rivaroxaban induces a significant downregulation of TGF-beta [9] and other proinflammatory cytokines [10], theoretically indicating its use prophylactically to prevent fibrotic and inflammatory closure of SVGs. As a result, the use of rivaroxaban may be indicated post-SVG and serve to decrease the risk of VGF in at-risk patients.

## 4. Conclusion

We used rivaroxaban instead of PCI for the treatment of SVG thrombosis presented as NSTEMI. This conservative approach worked in our patient due to his high clot burden being secondary to venous graft outflow mismatch. Rivaroxaban can be used as a safer, more effective long-term therapy for VGF thrombosis secondary to stasis and potentially indicated as a preventative measure for generalized VGF. Use of rivaroxaban in VGF may also serve to decrease the overuse of PCI in its treatment.

## Data Availability

No data were used to support this study.
